# Prevalence of smoking and high blood pressure, two major risk factors for non-communicable diseases: the SuRF NCD (surveillance of risk factors of non-communicable disease) report 2012

**DOI:** 10.15171/jcvtr.2016.36

**Published:** 2016-12-27

**Authors:** Ahmad Jamalizadeh, Zahra Kamiab, Ali Esmaeili Nadimi, Mohsen Nejadghaderi, Ala Saeidi, Amirhossein Porkarami

**Affiliations:** ^1^Department of Health, Rafsanjan University of Medical Science, Rafsanjan, Iran; ^2^Head of the Clinical Research Development Center, Department of Community Medicine, Rafsanjan University of Medical Science, Rafsanjan, Iran; ^3^Department of Cardiology, Rafsanjan University of Medical Science, Rafsanjan, Iran; ^4^Health Department, Rafsanjan University of Medical Science, Rafsanjan, Iran

**Keywords:** Hypertension, Smoking, Prevalence, Iran, Rafsanjan, SuRF NCD

## Abstract

***Introduction:*** In recent years non-communicable diseases (NCDs) risk factors such as tobacco consumption and high blood pressure (BP) have been increased. This study aimed to determine the frequency of risk factors of the main NCDs among inhabitants of Rafsanjan city.

***Methods:*** Our study is a part of NCD surveillance in Iran (SuRF NCD). A total of 640 people enrolled and divided in four age groups in urban and rural areas in Rafsanjan (a city in Kerman province). Data were collected using the standardized stepwise protocol for NCD risk factor surveillance of the World Health Organization (WHO). This study focused on hypertension (HTN) and smoking.

***Results:*** A total of 640 people (46.9% male and 53.1% female) participated in this cross-sectional study. The prevalence of HTN was 198 per 1000 population. 4.8% of those were below the age of 44, and 15% between 45 and 70 years old. Mean systolic BP was 127 ± 15.6 in male and 118 ± 19.65 in female and the statistical difference was significant (t = 5.55, *P* < 0.001). 79 (14.1%) of hypertensive live in urban and 32 (5.7%) live in rural areas (χ2 = 8.004, *P* = 0.005). The prevalence of current smokers was 112 per 1000 population; among them 56 (88.9%) were daily smokers. The mean age for starting smoking was 21.11 ± 7.16 years.

***Conclusion:*** Modifying risk factors such as HTN and smoking behavior through primary and secondary prevention programs by enhancing awareness and knowledge of lay people, improvement screening and treatment interventions, particularly for the youth is highly recommended.

## Introduction

Hypertension (HTN) is the cause of about 7.5 million deaths, approximately 12.8% of all deaths. It is a major risk factor for cardiovascular disease.^1^ The prevalence of HTN varies between 5.5% in rural India to 40% in some European countries. Iran is a multicultural and multi-ethnic country. The prevalence of HTN has been estimated about 22.1% among Iranian, but there is a notable variation in different geographical location, different ethnicities, socioeconomic status, different cultures and life style.^2,3^ Smoking is known as the single largest preventable risk factor for morbidity and premature death. It is the fourth risk factor attributable to burden of disease globally.^4^ Each year 6 million people die from tobacco use, both from direct and indirect consequences. By 2020, this number will grow to 7.5 million, accounting for 10% of all deaths. It is the cause of about 71% of lung cancer, 42% of chronic respiratory disease and 10% of cardiovascular disease.^1^ Tobacco epidemic is one of the greatest public health problems that human being has ever encountered.^5^ Tobacco use disproportionately affects males and lower socioeconomic groups in developed and developing countries.^6^ Approximately 80% of the more than 1 billion smokers worldwide live in low- and middle-income countries, where the burden of tobacco-related illness and death is heaviest.^5^

Although scattered research has done in Rafsanjan on non-communicable disease (NCD) and risk factors, but there was not comprehensive and updated study. Reliable evidence is the keystone for any NCD prevention plan to be initiated. We have done this research in urban and rural area among 640 inhabitants to investigate some risk factors of NCD.

## Materials and Methods

This cross-sectional study is a part of national survey on the prevalence of risk factors on NCDs, based on the World Health Organization (WHO) step by step instructions which has been done in the city of Rafsanjan, Kerman province in 2012.

The study population comprised 640 people aged 6 to 70 years old living in rural and urban areas of Rafsanjan city. It is located in north-west of Kerman. According to the census 2006, Statistical Center of Iran, its population was 295 175.^7^

This study was a probability proportional to size (PPS) multistage cluster sampling. We determined urban and rural population over 5 years old and 640 people enrolled, 370 urban and 270 rural. Interviewers were selected among health workers, technicians and experts. Interviewers were trained two sessions. Each survey team consists of one male and one female. The areas covered by each team and the number of households were previously specified. Each team was required to initially identify their families and with the permission and verbal consent, questioning was done.

### Blood pressure and smoking

The blood pressure (BP) was measured three times at the sitting position. There was a 5-minute rest between each measurement. A person’s BP was calculated as the average of the measurements. HTN was defined as systolic BP of at least 140 mm Hg and diastolic BP of at least 90 mm Hg, self-reported use of anti-hypertensive medications, or both. In addition, subjects were asked to state the number of cigarettes smoked per day.

### Demographic data

Demographic characteristics like age, marital status, and education were recorded by a trained interviewer. Ethnicity was determined by self-report and categorized as Persians, Turks, Kurds, and multi-ethnic.

Four age groups were considered for sampling: 6 to 14 years, 15 to 24 years, 25 to 44 years and 45 to 70 years. Questionnaires with any problems or deficiencies were sent back to be rechecked and completed.

### Statistical analysis

All the data were entered into the computer by one operator. Analysis was conducted using the SPSS version 20.0 by t test and analysis of variance (ANOVA) after check of normality by Kolmogorov‑Smirnov. A P value of less than 0.05 was taken as statistically significant.

## Results

A total of 640 people (46.9% male and 53.1% female) participated in this cross-sectional study. The mean age of respondents was 36.9 ± 17.39 years old (95% CI: 35.55-38.25). The mean age of men was 36.7 ± 18.09 and women 37.08 ± 16.7 years, and there was no significant difference between them (*P* = 0.78). Participants had a uniform cultural structure, comprising 99.5% Persians. The majority of participants were housekeepers (36.1%). Sociodemographic characteristic of the participants were presented in Table 1.

**Table 1:** Sociodemographic characteristics of respondents (n=640)

** Variables**	** No. (%)**
Gender	
Male	300 (46.9)
Female	340 (53.1)
Age	
6-14	79 (12.3)
15-24	103 (16.1)
25-44	234 (36.6)
45-70	224 (35)
Educational status	
Illiterate	102 (15.9)
Primary school	177 (27.7)
Secondary school	118 (18.4)
High school	150 (23.4)
University	93 (14.6)
Occupation	
Employee	45 (7)
Worker	25 (4.1)
House keeper	231 (36.1)
Self-employment	140 (21.9)
Student	138 (21.6)
Retired	34 (5.2)
Unemployment	27 (4.1)
Residential area	
Urban	270 (42)
Rural	370 (58)

BP of 561 participants was measured. 111 (19.8%) were hypertensive: 4.8% of those below the age of 44, and 15% between 45 and 70 years old. In general, the prevalence of HTN was 198 per 1000 population.

Mean systolic BP was 127 ± 15.6 in male and 118 ± 19.65 in female and the statistical difference was significant (t = 5.55, *P* < 0.001).

Mean diastolic BP in male and female was 79 ± 10.24 and 79 ± 11.76 respectively and there was no statistical difference (t = -0.153, *P* = 0.879).

Comparison of systolic and diastolic BP with ANOVA among different age group showed in Table 2. The mean systolic and diastolic BP increased with age in both sexes. At 15-24 and 25-44 age group mean systolic BP was significantly higher in male (*P* < 0.001 in both age group), but at 45-70 age group, it was not significant (*P* = 0.879).

**Table 2:** Comparison of systolic and diastolic blood pressure with ANOVA among different age group

** Average blood pressure**	** Gender**	** Age**	** Mean ± SD**	** F**	** P value**
Systolic blood pressure	Male	15-24	119±8.80	13.482	<0.001
		25-44	124±14.17		
		45-70	132±17.29		
	Female	15-24	105±12.14	75.62	<0.001
		25-44	112±12.95		
		45-70	132 ±20.61		
Diastolic blood pressure	Male	15-24	72±7.83	17.90	<0.001
		25-44	78±9.02		
		45-70	82±10.72		
	Female	15-24	72±9.48	43.11	<0.001
		25-44	77±9.27		
		45-70	86±12.02		

Mean diastolic BP at 45-70 age group in female significantly higher than male (*P* = 0.026), but there was no significant difference between both gender at other age groups.

With the χ^2^ test there was statistically difference in prevalence of HTN among urban and rural inhabitants.79 (14.1%) of hypertensive live in urban and 32 (5.7%) live in rural areas (χ^2^= 8.004, *P* = 0.005).

Fifty-seven (10.4%) was male and 54 (9.6%) female and there was no significant difference among both gender (χ^2^= 2.18, *P* = 0.139).

Characteristic of current smokers displayed in Table 3. Among 561 participants that asked about smoking, 63 (11.2%) were current smokers; among them 56 (88.9%) were daily smokers. In general the prevalence of current smokers was 112 per 1000 population. The mean age for starting smoking was 21.11 ± 7.16 years and there was no significant difference between both gender and residential area (t = -1.81, *P* = 0.075, t=-0.56, *P* = 0.57 respectively).

**Table 3:** Characteristics of current smokers

	** Category**	** No.**	**%**	** P value**
Current smoker	Female	4	0.7	<0.001
	Male	59	10.5
	Rural	36	6.4	0.786
	Urban	27	4.8
	15-24 years	0	0	<0.001
	25-44 years	20	3.5
	45-70 years	43	7.7	

Mean number of cigarettes smoked per day was 21.95 ± 17.05 and there was no significant difference among both gender and residential area rural and urban area (t = -0.866, *P* = 0. 391; t = -0.58, *P* = 0. 562 respectively).

## Discussion

In this study the prevalence of HTN was 19.8%, it was more prevalent among illiterate, 45-70 age group, and in urban area. There was no statistical difference in the prevalence of HTN between both sexes. There was no statistical difference in the mean of systolic and diastolic BP between both genders, but mean of diastolic BP was higher in older women and mean of systolic BP was higher in men under 45 years old.

The prevalence of HTN varies considerably among different cultures, ethnics, and populations.^8^ 18.0% of Ardabilian had raised BP and 3.7% had isolated systolic HTN, and it increased with age in both gender.^9^ these findings were consistent with our study. The prevalence of HTN almost consisted with other researchers conducted in East-Azerbaijan, Khorasan, Kohkilooyeh and Kerman, which prevalence of HTN in these provinces was16.29%, 17.74%, 21.31% and 20.65% respectively.^10-13^

In other cross-sectional study which included 3000 Isfahan citizens, overall prevalence of high BP was estimated 22.2%. The prevalence of high BP was greater in women than men (22.4% in comparison to 22.06%, *P* < 0.05) and increasing age was associated with risk of high BP (Odds ratio [OR] = 1.067, *P* < 0.01 ).^14^

Haghdoost et al in the systematic review estimated the overall prevalence of HTN around 23% in 30-55 year old population. The prevalence in men was 1.3% less than that in women (*P* < 0.0001). The mean diastolic BP in men was 0.62 mm Hg less than that in women while the mean systolic BP was 0.67 mm Hg greater,^3^ mean of diastolic BP was higher in older women and mean of systolic BP was higher in men less than 45 years old, which is almost, consist with this study.

In our study there was no statistical difference in the prevalence of HTN between sexes, the National Health and Nutrition Examination Survey (NHANES) showed no significant relation between gender and HTN among American adults between the years 1999-2004,^15^ in other studies in Iran, HTN was more frequent in male.^16,17^

In a study conducted in Rafsanjan in 2002, the prevalence of HTN was 23.4% among adult population, 25.1 of male and 22.4% of female had this problem. The age group of 70-79 years had the highest percentage of HTN. These results were almost similar with our research.^16^

We observed that 63 (11.2%) of participants were current smokers; among them 56 (88.9%) were daily smokers. The mean age for starting smoking was 21.11±7.16 years and there was no significant difference between both gender and residential area. It was most popular among men. Cigarette consumption is a stigma for women in Iranian culture. Thus there is probably some under-estimation about smoking by females. The highest prevalence of current cigarette smoking was in individuals aged 45-70 years (41%).

The prevalence of smoking in Sistan-Bluchestan and Bushehr was 20.3% and 21.1% respectively.^18^ In the national survey of risk factors of NCDs (SuRF NCD-2007) overall prevalence rate of current tobacco smoking (including cigarette smoking, water-pipe and pipe) was 14.8%, 26.1% for men and 3.2% among women. The prevalence of current cigarette smoking was 12.5%.^19^ Likewise Asgari et al reported 21.7% of male and 0.9% of female are every day smoker in our country.^20^

Khattab et al evaluated smoking habits in 10 countries of the Middle East and North Africa (MENA) region, among individuals aged ≥ 40 years. 31.2% of participants were current or past smoking of cigarettes. This proportion was significantly higher (*P* < 0.001) in men (48.0%) than in women (13.8%). Smoking rates were in general lowest in the Morocco (15.3%) and highest in Lebanon (53.6%).^21^

## Study limitations

A major limitation of our study was cross-sectional design; another limitation was small sample size, so there was the threat of limited generalizability.

## Conclusion

Our findings point to the impotence of modifying risk factors such as HTN and smoking behavior through primary and secondary prevention programs by enhancing awareness and knowledge of lay people, improvement screening and treatment intervention, and particular intervention for the youth.

## Competing interests

Authors declare no conflict of interest in this study.

## Ethical approval

Ethical issue of this study was approved in Vice Chancellor for Research Affairs of Faculty of Medicine, Rafsanjan University of Medical Sciences. Each participant was informed, prior the interview, about the purpose of the study and verbal informed consent was obtained from all participants. Also, the confidentiality of information was managed carefully by researchers.

## Acknowledgments

The authors would like to thank the Clinical Research Development Center for its support and collaboration in Ali Ebne Abitaleb hospital, Rafsanjan University of Medical Science, Rafsanjan, Iran.
